# A novel missense mutation of FOXC1 in an Axenfeld–Rieger syndrome patient with a congenital atrial septal defect and sublingual cyst: a case report and literature review

**DOI:** 10.1186/s12920-021-01103-w

**Published:** 2021-10-29

**Authors:** Kaiming Li, Min Tang, Manhua Xu, Yinggui Yu

**Affiliations:** Affiliated Hospital of Southwest Medical University, No.25, Taiping Street, Jiangyang District, Luzhou City, 646000 Sichuan Province China

**Keywords:** Axenfeld–Rieger syndrome, FOXC1, Mutation, Case report

## Abstract

**Background:**

Axenfeld–Rieger syndrome (ARS) is a rare autosomal dominant hereditary disease characterized primarily by maldevelopment of the anterior segment of both eyes, accompanied by developmental glaucoma, and other congenital anomalies. FOXC1 and PITX2 genes play important roles in the development of ARS.

**Case presentation:**

The present report describes a 7-year-old boy with iris dysplasia, displaced pupils, and congenital glaucoma in both eyes. The patient presented with a congenital atrial septal defect and sublingual cyst. The patient’s family has no clinical manifestations. Next generation sequencing identified a pathogenic heterozygous missense variant in FOXC1 gene (NM_001453:c. 246C>A, p. S82R) in the patient. Sanger sequencing confirmed this result, and this mutation was not detected in the other three family members.

**Conclusion:**

To the best of our knowledge, the results of our study reveal a novel mutation in the FOXC1 gene associated with ARS.

## Background

Axenfeld–Rieger syndrome (ARS, OMIM 602482) is an autosomal dominant hereditary disorder, involving malformation of the anterior segment of the eye during development and causing systemic malformations [1]. The incidence of ARS is estimated to be around 1:200,000 [2]. The major eye condition is ocular anterior segment dysgenesis (ASD), and approximately 50% patients secondarily develop glaucoma, which may induce blindness within a small number of years [3]. There are three types of ARS. Compared to the other two types, type 3 patients present more ocular characteristic and rarely present obvious dental nor facial abnormalities, but suffer from hearing loss and cardiac defects [1, 2, 4].

Two major developmental transcription factor genes, forkhead box C1 (FOXC1) on chromosome 6p25 and pituitary homeobox 2 (PITX2) on chromosome 4q25, have been demonstrated to cause the ARS disease, accounting for 40 to 70% of cases [5]. Individuals with PITX2 variants are more likely to have systemic features than individuals with FOXC1 variants, and mutations in the FOXC1 genes always associated with type 3 ARS [1, 6]. FOXC1 plays multiple roles in inter-charge mass spectrometry and organ development during normal embryogenesis, which are crucial for mesoderm, neurovascular, and eye development [7]. Herein, we describe the clinical features of a patient with ARS type 3 and the identification of a novel point mutation in the FOXC1 gene.

## Case presentation

**Fig. 1:**
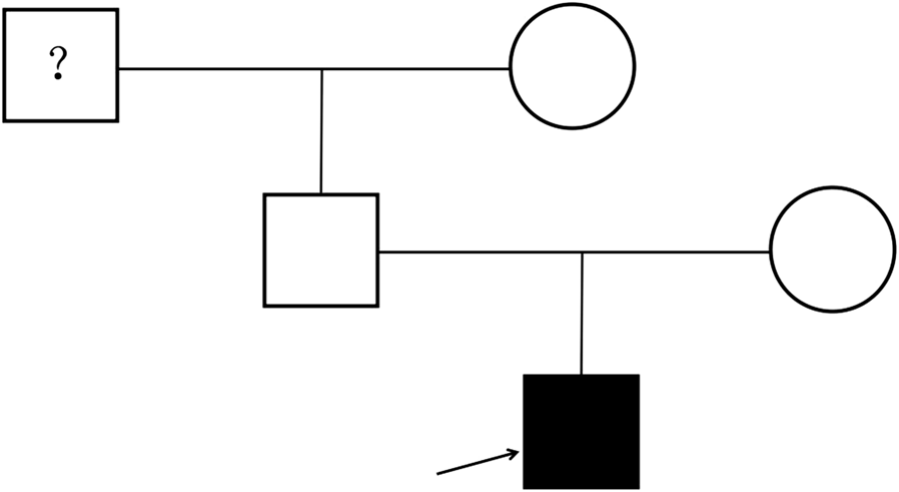
A family pedigree of three generations. The arrow indicates the patient

The patient was a 7-year-old boy in the southwest of China, who had blue cornea, photophobia, and poor vision from birth. When the subject was 36 days old, he had binocular anti-glaucoma surgery, which was followed by long-term treatment with travoprost and brinzolamide eye drops. However, the intraocular pressure (IOP) was not well controlled. The patient underwent surgeries to repair an atrial septal defect causing congenital heart disease and resection of a sublingual cyst. Recently, the eye symptoms worsened and the patient’s vision declined significantly. Thus, the boy and his family came to the Affiliated Hospital of Southwest Medical University. A three-generational pedigree of the patient’s family is shown in Fig. 1.

**Fig. 2:**
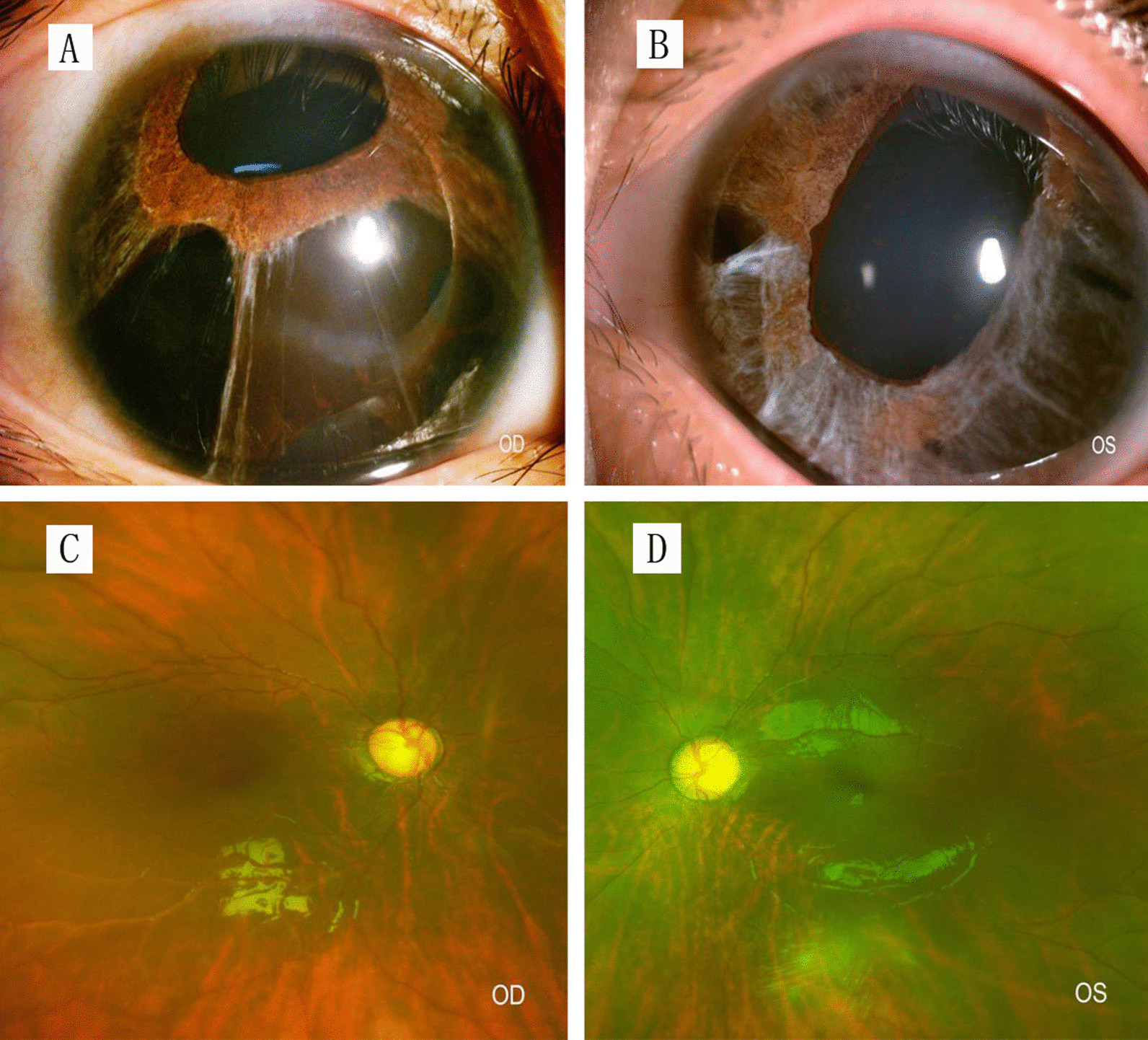
**A**, **B** The anterior segment of the eyes: the iris shows lamellar lacunae, polycoria, and corneal posterior embryotoxon. **C**, **D** Enlargement of the binocular cup to disc ratio indicates glaucoma-related optic disc damage

**Fig. 3:**
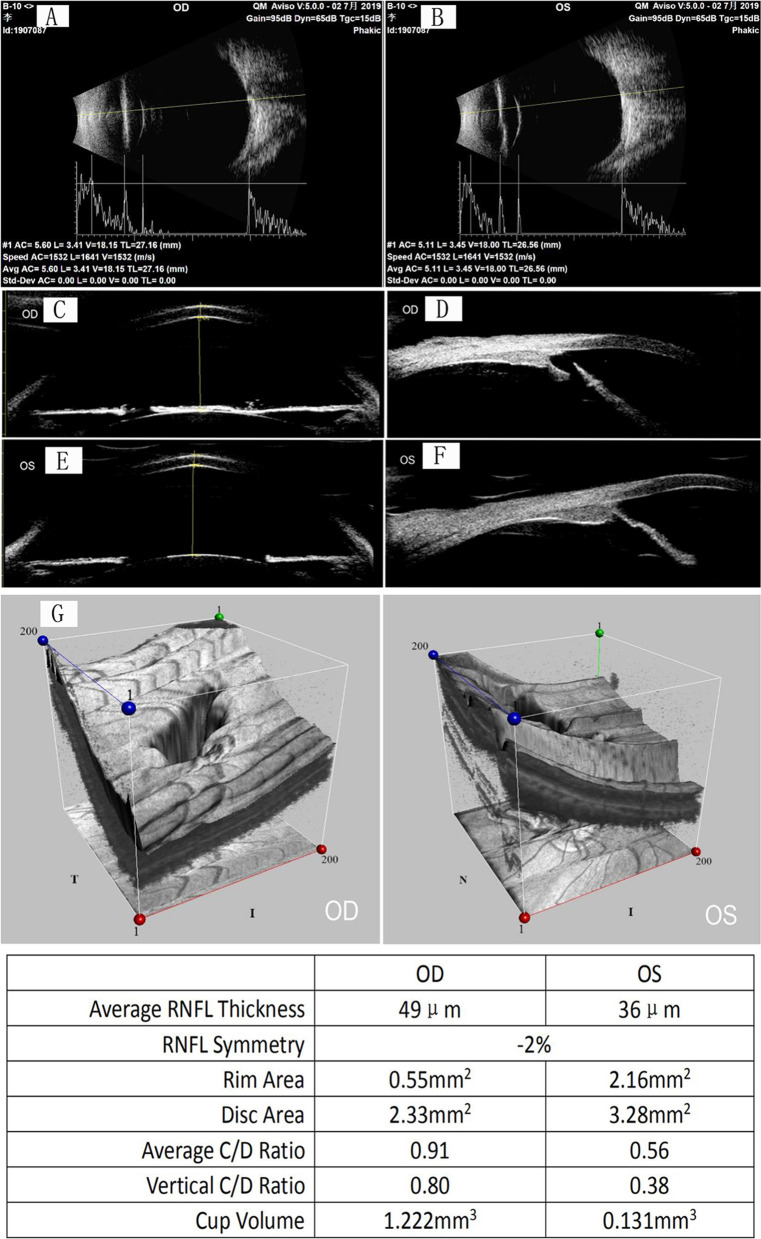
**A**, **B** Binocular B-ultrasound images show the prolongation of the axial axis. **C**–**F** Binocular UBM images show deepening of the anterior chamber, anterior adhesion, and fracture of the iris. **G** The OCT images of the binocular disc show that the binocular cup deepened and enlarged due to glaucoma damage

**Fig. 4:**
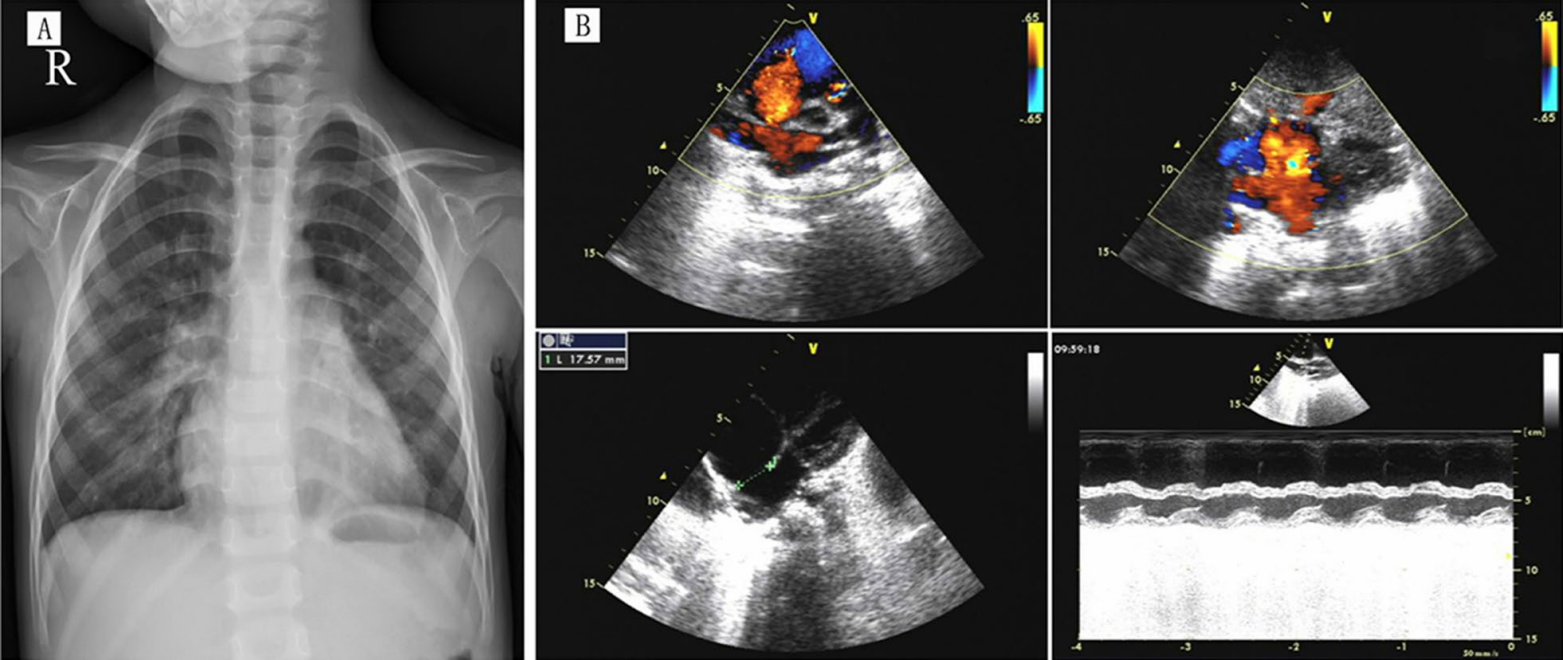
**A** The previous chest radiography from 4 years ago showed an enlarged podoid, a cardiothoracic ratio of about 0.52, and a slightly prominent left pulmonary artery. Preoperative echocardiography of the atrial septal defect showed the following two-dimensional measurements: RVOT: 24 mm; AOd: 16 mm; LA: 18 mm; AAO: 16 mm; LVDd: 27 mm; LVDs: 15 mm; IVS: 5 mm; LVPW: 5 mm; RV: 23 mm; MPA: 15 mm; RA: 31 × 37 mm; and HR: 107 b/m. Doppler measurements were AV: 1.04 m/s; MVe: 0.88 m/s; MVa: 0.53 m/s; PV: 1.32 m/s; TR: 2.7 m/s; and PG: 29 mmHg. **B** The diagnoses based on the ultrasound results was congenital heart disease, atrial septal defect (secondary foramen, central type), atrial horizontal left-to-right shunt, and tricuspid regurgitation (mild)

Ophthalmic examination of the patient revealed similar pathological changes in both eyes. Uncorrectable poor vision (VOD 0.08, VOS 0.06) and horizontal tremors were observed. No hyperemia or edema was detected in the conjunctiva. The cornea was transparent but the sclera was pale blue. The diameter of the cornea was 14 mm and the center of the anterior chamber was deep. The aqueous humor was clear. The texture of the iris was unclear with partial depigmentation. The lamellar and incomplete disconnections of the root of the iris formed some iridocyridosis and slit-like iris holes (from 4 to 9 o’clock in the right eye, 3 to 9 o’clock in the left eye) (Fig. 2A, B). The pupil displacement was worse in the right eye. The lens and vitreous of both eyes were slightly turbid. The optic disk was pale, with a cup to disk ratio of 0.9. The retina was ruddy with a wet-silk-like reflection, and the macula was not clear (Fig. 2C), D). IOP was 33 mmHg in the right eye and 20 mmHg in the left eye. Binocular B-ultrasound images showed prolongation of the axial axis (27.16 mm in the right eye, 26.56 mm in the left eye) associated with a long period of high IOP (Fig. 3A, B). Binocular ultrasound biomicroscopy (UBM) images showed deepening of the anterior chamber, anterior adhesion, and typical fracture of the iris (Fig. 3C–F). The optical coherence tomography (OCT) images of the binocular disk revealed a deepened binocular cup, which was enlarged due to the glaucoma damage (Fig. 3G). We also reviewed the patient's previous medical records, including chest X-ray images and cardiac color doppler ultrasound images (Fig. 4A, B).

According to the clinical manifestations, the patient was diagnosed with ARS (Type 3), bilateral congenital glaucoma, and bilateral congenital cataracts. To treat the glaucoma and relieve optic nerve atrophy, the patient underwent a trabeculectomy and iris circumferential resection in the right eye. After surgery, the IOP was 15 mmHg in the right eye. The IOP in the left eye dropped to 16 mmHg after treatment with anti-glaucoma drugs.

Considering the possibility of congenital disease, three of the boy’s family members (father, mother, and grandmother) underwent complete ophthalmologic and systemic examinations, but no positive findings were observed. Genetic tests were performed on the patient.

## Genetic analysis

This study was performed in accordance with the tenets of the Declaration of Helsinki. Approval to conduct this study was obtained from the Ethics Committee of Affiliated Hospital of Southwest Medical University (KY2019110). Informed consent was obtained from the guardian (parents), who agreed to join this study, and for the use of medical information and images for scientific research and publication.

With consent, all family members (patient, father, mother, and grandmother) had genetic testing. Peripheral blood from each family member was collected and sent to MyGenostics for testing. DNA samples from the patient were genetically tested using an NGS-based gene panel. For exome sequencing, we fragmented 1–3 μg of genomic DNA to an average size of 180 bp with a Bioruptor sonicator (Diagenode). Paired-end sequencing libraries were prepared using a DNA sample prep reagent kit (NEBNext). Library preparation included end repair, adapter ligation, and polymerase chain reaction enrichment. These procedures were performed following Illumina protocols. Amplified DNA was captured using a Gencap Capture kit (MyGenostics GenCap Enrichment technologies) containing probes for 50 genes related to iris disease. DNA probes were designed to tile along the exon regions of genes related to iris diseases. The enrichment libraries were sequenced on Illumina Nova 6000 sequencer for a paired read of 150 bp (average sequencing depth: 746.71; target area coverage: 10X: 99.80%, 20X: 99.68%). After sequencing, the raw data were saved as a FASTQ format and bioinformatics analyses were performed.

**Fig. 5:**
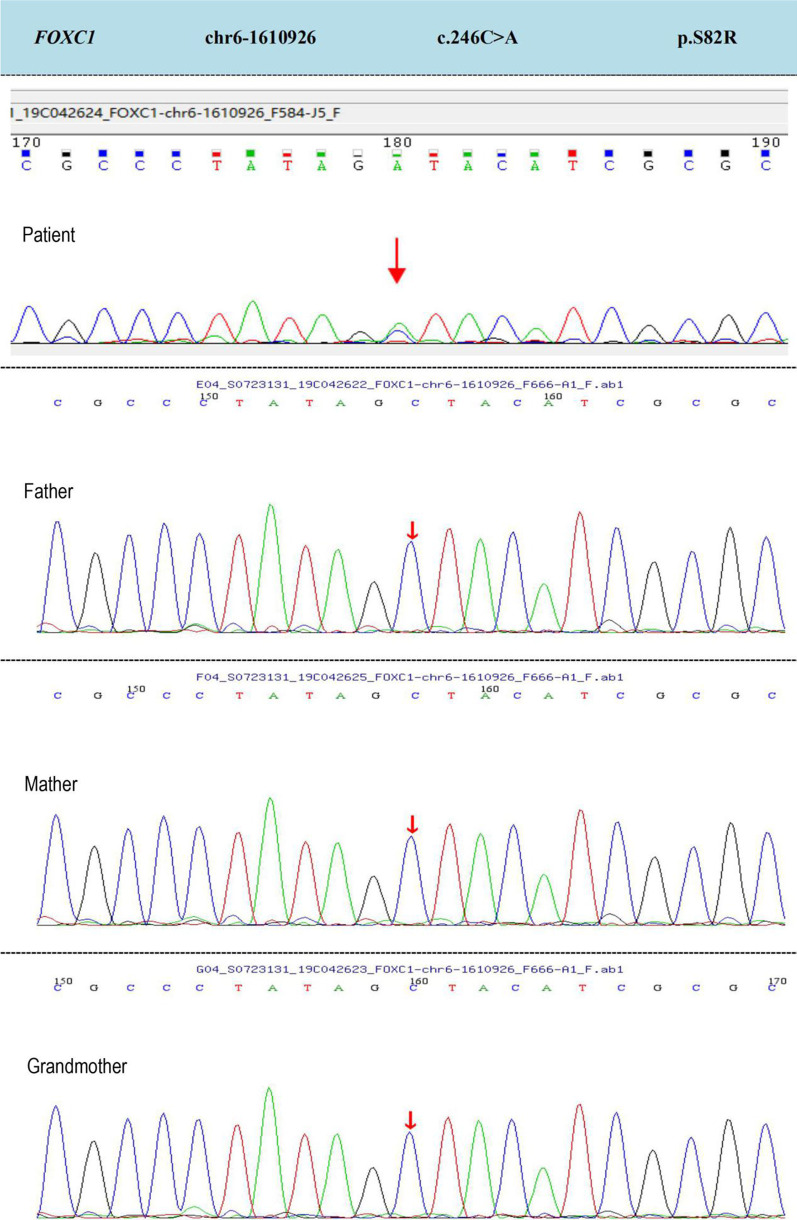
Detection and comparison of the genes show that the mutation site of the patient was NM_001453:c.246C>A. The patient had a de novo heterozygous mutation with autosomal dominant inheritance. No FOXC1 mutation was found in other family members

**Fig. 6:**
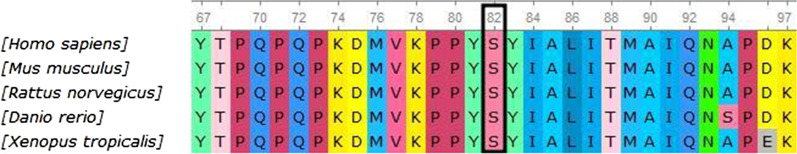
Amino acid sequence alignment of FOXC1 from different vertebrates. Black rectangle indicates that serine-82 is highly conserved in these species

After removing the low-quality reads (< 80 bp), contamination and linkers from the raw data, the clean reads were mapped to the UCSC hg19 human reference genome using BWA software. The single nucleotide variations (SNVs) as wells as inserts and deletions (INDEL) were identified using GATK software, followed by annotation using ANNOVAR software. The mutations with minor allele frequencies (MAF) less than 0.02 were excluded, which included the 1000 Genomes Project, ESP6500, EXAC and gnomAD databases. The pathogenicity and conservatism of missense mutations were predicted using SIFT, PolyPhen-2, MutationTaster, GERP++ and REVEL. The pathogenicity of splice site changes was analyzed using SPIDEX. Finally, four suspected pathogenic variants were identified. Among them, three recessive heterozygous variants, CEP290 (exon50, NM_025114:c.6869dupA), ELP4 (exon10, NM_001288726:c.1315C>T), and OCA2 (exon7, NM_000275:c.797G>A), were not associated with the present clinical phenotype and were excluded (Table 1). One recessive heterozygous variant in FOXC1 (exon1, NM_001453:c. 246C>A, p. S82R) closely related ARS (type 3) was identified in this proband (Fig. 5, Table 2). Protein prediction revealed that the cytosine to adenine mutation at nucleotide 246 caused a serine to arginine change at amino acid 82 in FOXC1. SIFT, PolyPhen-2, MutationTaster, GERP++ and REVEL which scored 0.000, 0.999, 0.999, 2.98, 0.968 respectively, all predicted c. 246C>A as “disease causing”. The variation interpretation guideline of American College of Medical Genetics (ACMG) also predicted the variant as “pathogenic”, causing ARS (Type 3). Additionally, The c. 246C>A mutation in FOXC1 with ARS was found not to be reported in gnomAD, HGMD, Clinvar and dbSNP databases. Amino acid sequence alignment of FOXC1 across species indicates evolutionary conservation at serine-82 (Fig. 6). The DNA samples of the patient’s family members were sequenced (targeted Sanger sequencing) to validate the variation in the targeted exome FOXC1 (Fig. 5, Table 2). The c. 246C>A mutation was not found among the family members.

**Table 1:** The proband’s three recessive heterozygous genes

Gene	Chromosomal Location	Transcriptional exons	Nucleotides and amino acids	Homozygous/heterozygous	Normal frequency	Calculate	Pathogenicity analysis	Mode of inheritance	The phenotype of diseases
*CEP290*	chr12-88449443–88449443	NM_025114;exon50	c.6869dupA (p.N2290K fs*6)	Het	0.00010	–	Pathogenic	1. AR 2. AR 3. AR 4. AR 5. AR	(1) Bardet-Biedl syndrome (Type 14); (2) Joubert syndrome (Type 5); (3) Leber's congenital Haimeng (Type 10); (4) Meckel syndrome (Type 4); (5) SeniorLoken syndrome (Type 6)
*ELP4*	chr11-31703506	NM_001288726;exon10	c.1315C>T(p.L439F)	Het	0.01140	B	Uncertain	AD	Aniridia (Type 2)
*OCA2*	chr15-28263553	NM_000275;exon7	c.797G>A(p.R266Q)	Het	0.00080	B	Uncertain	1. AR 2. AR	(1) Albinism (Type II); (2) Skin/hair/eye pigment variant (Type 1)

Prediction: protein function prediction software REVEL (rare exome variant ensemble learner); P, prediction is harmful; B, prediction is benign; –, unknown

**Table 2:** Genetic variation highly related to the clinical phenotype of the subjects

Gene	Chromosomal Location	Transcriptional exons	Nucleotides and amino acids	Homozygous/heterozygous	Normal frequency	Calculate	Pathogenicity analysis	Mode of inheritance	The phenotype of diseases	Source of variation
FOXC1	chr6-1610926	NM_001453;exon1	c.246C > A(p.S82R)	Het	–	P	Likely pathogenic	1.AD 2.AD	1.Axenfeld–Rieger syndrome (Type 3); 2.Iridodystrophy (Type 1)	de novo mutation

Prediction, protein function prediction software REVEL (rare exome variant ensemble learner); P, prediction is harmful; B, prediction is benign; –, unknown

## Discussion and conclusions

ARS is a rare, genetic disease affecting multiple organ systems. Previous studies have reported that patients with ARS showed a variety of overlapping phenotype of ocular and systemic clinical features [1, 2, 4]. By reviewing literatures [3, 4, 8, 9], the ocular phenotypic characteristics we found in ARS include glaucoma, hypertelorism, iridogoniodysgenesis, peripheral anterior synechiae, corectopia, polycoriaposterior embryotoxon, and ectropion uveae. Common systemic features observed in ARS patients include craniofacial dysmorphism (e.g. prominent forehead, telecanthus, and midface hypoplasia), dental anomalies (e.g. hypodontia, microdontia, oligodontia, and adontia), sensorineural deafness, cardiac abnormalities (e.g. heart valve malformations, atrial septal defect) and a redundant periumblical skin. By contrast, Peters anomaly, kidney abnormalities, hydrocephalus, growth retardation, variable neurological and skeletal anomalies are less common. Based on typical ocular and systemic features, the patient in the present study was identified as ARS, although hearing loss was not observed. The sublingual cyst was not a congenital disease, which was a rare concomitant complication in previous cases. However, the histological origin of the sublingual cyst was mesoderm which is closely related to ARS development. Therefore, it may be a new phenotype of ARS. The management of individuals affected by ARS requires a multidisciplinary approach, including dedicated surveillance and treatment of glaucoma, sensorineural hearing loss, and cardiac, endocrinological, craniofacial, and orthopedic abnormalities [10]. The present patient underwent heart repair surgery when he was very young with good results. But unfortunately, the glaucoma was not well controlled, even though he had anti-glaucoma surgery at a very early age. Therefore, early diagnosis and treatment of glaucoma is the most difficult problem in the disease.

FOXC1 is a single exon gene that codes for a member of the large forkhead box transcription factor family. ARS shows genetic heterogeneity, and the clinical phenotypic features of ARS patients are with incomplete penetrance and variable expressivity. The phenotype of ARS varies considerably among cases, and even between the two eyes of the same patient [11–13]. FOXC1 is the most widely studied gene in ARS, and several studies have characterized the molecular consequence of various FOXC1 mutations. Here, we summarize the common FOXC1 variations causing ARS according to recent reports, in which the representative mutations are described (Table 3). To date, more than 80 different FOXC1 mutations have been detected in ARS patients [1, 14, 15]. These data suggests that the most common FOXC1 defects leading to ARS are point mutations, especially missense mutations, which was also observed in our patient. The c.246C>A was segregated in the proband with typical ARS phenotype, while not in other family members. Therefore, we conclude that it is de novo. Previous reports also indicate that duplication mutations of FOXC1 were found primarily present with iris hypoplasia and glaucoma, while those with missense mutations have various manifestations with extraocular phenotypes. Most of these cases are caused by heterozygous mutations in FOXC1, a few by homozygous mutations [9]. Furthermore, although several genes have been found to cause the condition, the etiology of many ARS patients is still unknown, indicating the involvement of posttranslational modification and the effect of epigenetics [15]. Additionally, accurate determination of the proportion of ARS cases caused due to FOXC1 mutations are difficult to calculate, since ARS is a rare genetic disease and phenotypic severity is so variable.

**Table 3:** Representative clinical manifestations attributed to the FOXC1 mutations with ARS

cDNA variant	Amino acid variant	Mutation type	Ocular		Systemic				Other	Reference
			ASD	Glaucoma	Facial/Dental	Cardiac	Hearing	Umbilical		
c.246C>A	p.S82R	Missense	+	+	+	+	−	−	Sublingual cyst	The present study
c.335T>C	p.Phe112Ser	Missense	+	+	+	+	NR	NR	Peters anomaly	[12]
c.454T>C	p.Trp152Arg	Missense	+	+	+	NR	+	NR	NR	[9]
c.358C>T	p.Q120X	Nonsense	+	+	+	+	NR	+	Peters anomaly, urethral malformations	[16]
c.380T>G	p.R127L	Missense	+	+	−	+	NR	NR	NR	[17]
c.508C>T	p.Arg170Trp	Missense	+	+	+	+	+	−	NA	[18]
c.161T>A	p.M161K	Missense	+	+	+	NR	NR	NR	NR	[19]
c.192 C>G	p.Tyr64Ter	Missense	+	+	+	−	NR	−	Growth retardation	[20]
c.4C>T	p.Q2X	Missense	+	+	NR	NR	+	NR	NR	[21]
c.272T>C	p.Ile9lThr	Missense	+	+	NR	NR	NR	NR	NR	[14]
c.317delA	p.Gln106Argfs*75	Nonsense	+	+	−	−	−	−	−	[11]
c.477C>G	p.Tyr159*	Nonsense	+	+	NR	NR	NR	NR	Leukoencephalopathy, Growth retardation	[22]
c.407_409delGTC	p.V137del	Nonsense	+	+	+	−	−	−	−	[23]
c.477_478delCA	p.(Y159 ∗)	Frameshift	+	+	−	NR	+	NR	NR	[15]
c.210_210delG	p.Q70Hfs*8	Deletion	+	+	NR	+	NR	NR	NR	[17]
c.1494delG	p.G499Afs*20	Deletion	+	+	+	NR	NR	−	NR	[3]
c.92_100del	p.Ala31_Ala33del	Deletion	+	+	−	NR	−	NR	NR	[9]

ASD, anterior segment dysplasia; +, positive; −, negative; NR, not reported

Here, we first report a de novo heterozygous missense mutation (NM_001453:c. 246C>A, p. S82R) in FOXC1 in a patient with ARS type 3, and our report may be useful for better understanding of the spectrum of FOXC1 mutations and provides further information about the phenotypic characteristics of ARS patients. Furthermore, the hybrid recessive mutations of CEP290, ELP4, and OCA2 were also detected in the proband, but may have nothing to do with the clinical phenotype. However, further study is needed.

## Data Availability

The details of the variant analysed during the current study are available in the ClinVar database repository, under the accession number SCV001870394 (https://www.ncbi.nlm.nih.gov/clinvar/variation/1251979/). The raw datasets generated and/or analysed during the current study are not publicly available in order to protect participant confidentiality but are available from the corresponding author on reasonable request. Public databases used in this study included Human reference genome (GRCH37/hg19) (https://www.ncbi.nlm.nih.gov/assembly/GCF_000001405.13/), 1000 genomes database (http://www.1000genomes.org/), EXAC (http://exac.broad institute.org/), gnomAD (http://gnomad.broadinstitute.org/about), and dbSNP (http://www.ncbi.nlm.nih.gov/projects/SNP/).

## Abbreviations

ARS: Axenfeld–Rieger syndrome
NGS: Next generation sequencing
ASD: Anterior segment dysgenesis
FOXC1: Forkhead box C1
PITX2: Pituitary homeobox 2
IOP: Intraocular pressure
UBM: Ultrasound biomicroscopy
OCT: Optical coherence tomography
SNVs: Single nucleotide variations
INDEL: Inserts and deletions
MAF: Minor allele frequencies
ACMG: American College of Medical Genetics
